# Whispering-gallery nanocavity plasmon-enhanced Raman spectroscopy

**DOI:** 10.1038/srep15012

**Published:** 2015-10-07

**Authors:** Jing Zhang, Jinxing Li, Shiwei Tang, Yangfu Fang, Jiao Wang, Gaoshan Huang, Ran Liu, Lirong Zheng, Xugao Cui, Yongfeng Mei

**Affiliations:** 1 Department of Materials Science, Fudan University, Shanghai 200433, People’s Republic of China; 2 Department of Light Sources & Illuminating Engineering, School of Information Science & Technology, Fudan University, Shanghai 200433, People’s Republic of China; 3 School of Information Science & Technology, Fudan University, Shanghai 200433, People’s Republic of China

## Abstract

The synergy effect in nature could enable fantastic improvement of functional properties and associated effects. The detection performance of surface-enhanced Raman scattering (SERS) can be highly strengthened under the cooperation with other factors. Here, greatly-enhanced SERS detection is realized based on rolled-up tubular nano-resonators decorated with silver nanoparticles. The synergy effect between whispering-gallery-mode (WGM) and surface plasmon leads to an extra enhancement at the order of 10^5^ compared to non-resonant flat SERS substrates, which can be well tuned by altering the diameter of micron- and nanotubes and the excitation laser wavelengths. Such synchronous and coherent coupling between plasmonics and photonics could lead to new principle and design for various sub-wavelength optical devices, e.g. plasmonic waveguides and hyperbolic metamaterials.

Sensors and detectors that are miniature and ultra-sensitive have been a dream of researchers in nanoscience. Surface plasmons are able to achieve nanoscale confinement of electromagnetic fields, which has led to one of the most promising platforms for sensing^1^,^2^,^3^,^4^,^5^,^6^. Among various plasmonic nanosensors and detectors, the surface enhanced Raman scattering (SERS) is a powerful spectroscopy technique that can provide fingerprint, non-destructive and ultra-sensitive characterization^7^,^8^,^9^,^10^,^11^. SERS is intrinsically considered as a nanostructure-based phenomenon, as the intensity of the Raman scattered light crucially depends on size, shape and inter-particle spacing at nanoscale. Tremendous SERS substrates are designed by tailoring components and configurations of plasmonic structures into a two-dimensional array^12^,^13^,^14^,^15^,^16^,^17^,^18^,^19^. Principally, the sensitivity of SERS sensors is ultimately determined by the quality factor of plasmon resonance^20^,^21^,^22^. However, the intrinsically high ohmic losses of metal result in low resonance quality factors, thus limiting their enhancement and sensitivity. Therefore, combining SERS with special optical structures (for example: multihole capillaries, photonic crystals and WGM cavities) for optimizing comes as a preferred method^23^,^24^,^25^,^26^. Optical cavities with WGMs, which confine light to small volumes by resonant photon circulation could greatly enhance the light-matter interaction^27^,^28^,^29^,^30^,^31^,^32^,^33^. Ultra-sensitive detection has been demonstrated by resolving the small changes in the whispering-gallery resonating spectrum induced by the nanoscale objects^34^,^35^,^36^,^37^,^38^,^39^,^40^. Unfortunately, fingerprinting spectroscopic measurement is still challenging to be achieved in such dielectric optical cavities. As a coupling effect with the combination of plasmon and whispering-gallery resonance, it has recently been proposed that the quality factor of surface plasmon polariton can be greatly enhanced by on a high-Q silica microresonator^41^. With proper geometrical structure and fabrication methods, the radiation and scattering loss can be ideally suppressed and minimized.

Self-assembly concepts inspired from natural contexts could lead to various advances in engineering materials with complex geometrical structures^42^. One particularly useful form of self-assembly involves rolling and folding two-dimensional materials into three-dimensional (3D) cavities with nanoscale dimension and specific functions^43^,^44^,^45^. In this work, a strain-engineered self-rolling process is used to build a new Raman-enhanced sensor that synchronously and coherently supports both plasmonic and whispering-gallery resonances in a nanocavity. Figure 1a presents a schematic image of the device. It consists of a tubular nanocavity formed the rolling up of strained SiO/TiO_2_ (3/3 nm) dielectric multilayers, which are fully and uniformly decorated with Ag metallic nanoparticles (NPs, diameter: ~25 nm). This multilayer combination is selected based on the optical properties and the scalability of strain-engineered 3D structures. Guided rolling and self-assembly of the Ag NPs are achieved by a one-step thermal annealing process which releases the strained SiO/TiO_2_/Ag thin films from a polymer sacrificial layer to form the rolled-up nanotube while simultaneously triggering the dewetting-induced self-assembly of the Ag nanodroplets from the Ag thin film (see Methods). A scanning electron microscope (SEM) image of one of the fabricated devices is shown in Fig. 1b. The surface plasmon resonance between the dewetted Ag NPs is thus transferred to enhance the Raman signals of chemicals on the cavity walls. These rolled-up optical cavities with whispering-gallery modes (WGMs) provide extremely low photon loss rate and small cavity mode volumes for localization and concentration of the electromagnetic field.

We first calculate the WGMs in the nanocavity based on Mie scattering theory, which is deterministically related to plasmonic resonance. Here, the nanomembrane layer is treated as a silver NP-air composite layer with a thickness of 25 nm (average NP size) on the top, and a SiO/TiO_2_ layer with thickness of 3/3 nm at the bottom. Maxwell-Garnet effective media theory (EMT) is introduced to describe the interaction between the incident light and the nanomembrane layers for our plasmon nanocavities^46^. The effective refraction index is calculated by taking into account the material dispersion of both the silver NPs and dielectric SiO/TiO_2_ thin films (see Supplementary Notes, Part 1). Figure 1c shows the average electric field intensity over the wall of the tubular cavity as functions of the excitation laser wavelength (vertical axis) and the tube diameter (horizontal axis). The calculated results reveal that light with certain wavelength can circulate repeatedly in the nanocavity with desired diameters, which results in a highly enhanced local electromagnetic field around the cavity walls. The calculated mode volume is only 3.16 lambda^3^,^47^. The large surface-to-physical-volume ratio of the nanocavity strengthens the surface localization effect for exhibiting well-confined strong local fields. According to the electromagnetic mechanism (EM) using classic electro-dynamics methods, the Enhancement factor (EF) of Raman intensities can be written as under the Mie theory^48^


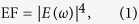


where *E*(*ω*) is the intensity of the electrical field for the light with frequency *ω*. Thus we believe that the WGMs are able to exhibit well-confined strong local fields around tubular cavity walls (where molecules are attached), dramatically amplifying the Raman signals when compared to 2D surface plasmon resonators.

To evaluate the Raman enhancement capacity and the sensitivity of the device, we test the device in different imaging configurations. A rolled-up tubular plasmon cavity with a diameter of 850 nm is fabricated and imaged by SEM (Fig. 2a). Three regions with different morphologies, including Ag NPs array on SiO/TiO2 nanomembrane (in the left side of plamsmon nanocavity), tubular plamsmon nanocavity and silicon substrate (in the right side of plamsmon nanocavity), are clearly shown. Rhodamine solution (R6G, 10^−5^ M), which was used as a probe chemical here, was subsequently dropped into the device area and dried in air for the Raman measurement. A 514.5-nm laser was used to excite the Raman spectra of R6G, and a 2D micro-Raman mapping corresponding to an area of 3.2 × 1.8 μm^2^ was acquired. The color-coded Raman mapping in Fig. 2b shows the peak intensity at 1364 cm^−1^ of R6G in different positions. It is observed that the three areas (dark red, bright yellow and dark black from the left to the right) of the Raman intensity map exactly correlates with three regions of the sample in the SEM (Fig. 2a), which indicates an extra enhancement of Raman intensity for R6G on the nanocavity. Detailed Raman spectra at different featured locations along the green arrow in Fig. 2a are displayed in Fig. 2c. On the bare silicon substrate, no Raman signal can be detected, while Raman spectra of R6G are obtained on the 2D NP array. It indicates that surface plasmon modes in silver nanoparticles are excited and contribute to the enhancement of Raman scattering (i.e. SERS). As for the spectra from plasmonic tubular nanocavity, the SERS effect is remarkably enhanced, as shown in the red spectra of Fig. 2c. Such SERS enhancement on plasmon nanocavity suggests that besides surface plasmon effect of silver nanoparticles, the tubular geometry which supports WGMs could greatly improve SERS due to a coupling effect. We then quantitatively explore the magnitude of Raman enhancement in the tubular nanocavities. A series of R6G solutions with various concentrations from 10^−1^ to 10^−13^ M were prepared and measured on three different kinds of substrates. The lowest detectable concentration for R6G solution on each substrate and the corresponding Raman spectra are revealed in Fig. 2d. For the NP-decorated plasmonic nanocavities, the detection limit can be as low as 10^−12^ M (spectra I in Fig. 2d). Interestingly, rolled-up TiO_2_/SiO nanocavities without silver NPs (spectra II in Fig. 2d) also provide an enhanced Raman detection limit (down to 10^−7^ M R6G solution) compared to flat nanomembranes without silver nanoparticles (spectra III in Fig. 2d). Our calculation shows that the EF of Raman signal in plasmonic nanocavities approach the order of 10^10^. (See detailed results and calculations in Supplementary Notes, Part 2)

For the purpose of demonstrating the tunability of WGMs as well as the Raman enhancement factor of the nanodevice, a series of Raman measurements was performed on a conical plasmon nanocavity with diameters ranging from 242 to 1030 nm (Fig. 3a, right vertical axis) along a conical tube. The theoretical result based on Mie-scattering as a function of tube diameter is also presented by the red line in Fig. 3a, while the left vertical axis stands for the averaged quadruplicate electric field intensity in the tube wall. Figure 3b shows the finite-difference time-domain (FDTD) simulation of |E|^2^ distribution in the tubular plasmon nanocavity at the excitation wavelength of 514.5 nm. The size-dependent enhancements in Fig. 3a,b do not show a monotonic increase with increasing size of the nanocavities, but show a series of peaks at diameters of ~500 and 820 nm. The normalized Raman signal peak intensity at 1364 cm^−1^ of R6G measured from the conical plasmonic nanocavity (blue circles) agrees well with the theoretical estimations, while much stronger Raman signals were obtained at the positions with diameters of 490 and 850 nm (Fig. 3a). FDTD simulations from Fig. 3c demonstrate that the enhancement at the tube diameter of ~500 nm mainly originates from the resonant mode with azimuthal number *m* = 3, while the enhancement at the tube diameter of ~820 nm mainly results from the resonant mode with azimuthal number *m* = 5.

The tunability of WGMs in the nanocavity is also achieved by using different excitation wavelengths. The experimental results obtained by using a 632.8-nm laser excitation are shown in Fig. 3e by the blue circles, where the strongest Raman intensity moves to the diameter of 240 and 695 nm subsequently along a conical tube. Theoretical calculation and FDTD simulation of the of electric field distribution in the tubular plasmonic nanocavity by using a 632.8-nm laser excitation are also displayed in Fig. 3e and Fig. 3f, respectively. Both theoretical and experimental data agree with each other and confirm our proposed coupling effect between WGMs and plasmon resonance. The theoretical maximal enhancement at the diameter of ~240 nm mainly corresponds to the resonant mode with *m* = 1. The above results under two excitation laser lines (514.5 and 632.8 nm) demonstrate the Raman enhancement in the plasmon nanocavity is highly tunable by the laser wavelength and the cavity diameter.

Conical nanocavities with gradually changing diameters are fabricated by the strain-engineered self-rolling method (see Methods). Figure 3g displays a SEM image of a conical nanocavity with a diameter changing from 150 to 900 nm. Micro-Raman mapping is performed on such conical nanocavity to evaluate the Raman enhancement in different position. Figure 3h displays the microscopy image of the conical plasmon nanocavity, while the mapping Raman intensity of the 1650 cm^−1^ band of R6G molecules excited by a 514.5-nm laser is presented in Fig. 3i. Similar to Fig. 2c, the intensity of the Raman signal correlates with the position of the conical nanocavity (Fig. 3j). Remarkably, we see a series of Raman signal maximums along the axis of the cone, while ~4.2 μm space between the maximums is clearly measured. As expected, such peaks mainly originate from the resonant modes with azimuthal numbers *m* = 1, 3 and 5. Since the WGMs in the nanocavity are strongly dependent on the cavity diameter, such a conical cavity with a changing geometric parameter could form a self-tuning resonator for excitation by a laser with an arbitrary wavelength. In other words, for any chemical molecules in the nanocavity, using an appointed incident light, its absorption can be enhanced at the resonating point of the conical nanocavity, leading a greatly amplified Raman signal.

Due to the advances in the strain-engineered self-rolling method, the nanocavity can reach a length of almost several centimeters and still maintain good uniformity in nanoscale diameter. Figure 4a displays a long and uniform rolled-up plasmonic nanocavity. The device thus could be easily integrated with other nanosystems, for example, to be transferred as an active nanoscale optofluidic sensor, as schematically illustrated in Fig. 4b. Such a combination enables an ideal sensing platform for trace chemical detection with several advantages, including fingerprinting, ultra-sensitivity and minimal use of samples/reagents. We show that this approach could be used for inspecting pesticide residues with ultra-small sample volumes. A liquid sample of the pesticide parathion solution is easily pumped in the nanocavity by the capillary effect. Figure 4c, corresponding to Supplementary Video, displays the solution of pesticide parathion pumped into the nanocavity. The calculated sample volume is only 0.02 pL. Figure 4 reveals that normal Raman spectra recorded on flat Ag NPs array using methanol (curve I) and water as solvent (curve II). No obvious band is observed by shedding the laser on the solution dispersed on the flat Ag NPs array. By pumping the sample solution into the plasmonic nanocavity, we can clearly detect two bands at 1,590  and 1,341 cm^−1^ (curve III and cure IV) that are characteristic bands of parathion residues^11^,^49^. This demonstrates that such tubular plasmonic nanocavity could have tremendous scope as a simple-to-use, field-portable and cost-effective nanofluidic analyzer or single cell nanoprobe.

We have demosntrated that the unique geometry of the whispering-gallery plasmonic nanotubular cavities, which are fabricated by a strain-engineered self-rolling approach, can significantly enhance the surface plasmon resonance as a result of highly concentrated optical fields. Such synchronous and coherent coupling of the plasmonic and whispering-gallery resonance greatly enhances Raman signals, which could potencially be a simple and robust methodtowards single-molecular detection with good optimization. The tunability of the coupling effect could open up novel ways to develop new device concepts for high performance deep-sub-wavelength optoelectronic devices such as plasmonic waveguides, single cell photonic nanoprobes, and hyperbolic metamaterials.

## Methods

### Fabrication of plasmon nanotubes with Silver NPs

PMMA (5 wt% in acetone) was first spin-coated on silicon substrate as the sacrificial layer, and SiO/TiO_2_/silver were deposited in sequence by electron beam evaporation onto the PMMA layer. Rapid thermal annealing (ULVAC ACS-4000-C4) in nitrogen process was followed afterward to release the pre-strained SiO/TiO_2_/silver nanomembrane and to form silver NPs. The annealing temperature was 600 °C and the annealing time was 40 s. Morphologies of nanotubes and NPs were characterized by scanning electron microscope. More detailed information about the influences of silver NPs on the forming of nanotubes can be seen in Supplementary Note 3.

### Raman measurement

R6G in deionized water solutions with concentrations range from 10^−1^ M to 10^−13^ M were prepared for the Raman measurement. R6G solution with a volume of about 0.5 mL was droplet on the substrate (1 × 1 cm) and dried in air. Raman spectra were acquired by micro-Raman Spectrometer (Renishaw InVia RM 1000) with incident wavelengths of 514.5 and 632.8 nm. The laser power was the same through the whole measurement process, and the accumulation time was 10 s.

## Additional Information

**How to cite this article**: Zhang, J. *et al*. Whispering-gallery nanocavity plasmon-enhanced Raman spectroscopy. *Sci. Rep*. **5**, 15012; doi: 10.1038/srep15012 (2015).

## Supplementary Material

Supplementary Information

Supplementary Video

## Figures and Tables

**Figure 1:**
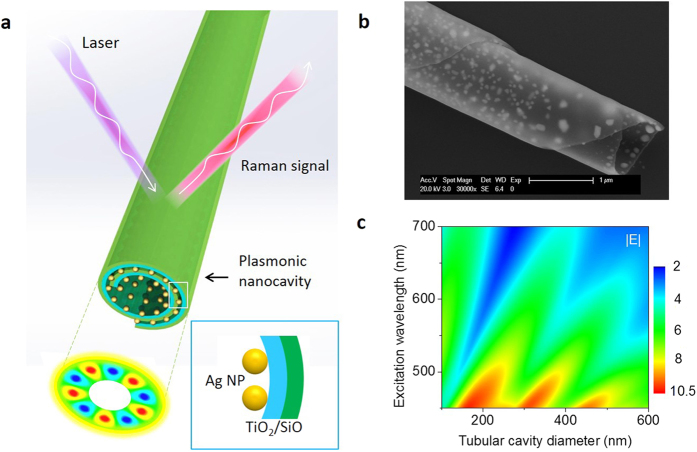
Rolled-up whispering-gallery plasmon nanocavity. (**a**) Schematic configuration of an whispering-gallery plasmon nanocavity rolled-up from a strained AgNP/TiO_2_/SiO multiple nanomembranes. (**b**) Enlarged SEM image at the end of the fabricated tubular plasmon nanocavity (thickness: TiO_2_ 3 nm, SiO 3 nm; Ag NP diameter: ~25 nm). The scale bar is 1 μm. (**c**) Calculated average electric field intensity |E| over the tube wall of the whispering-gallery plasmon nanocavity as functions of the cavity diameter and the excitation wavelength. The E-filed is the relative value normalized to excitation field intensity.

**Figure 2:**
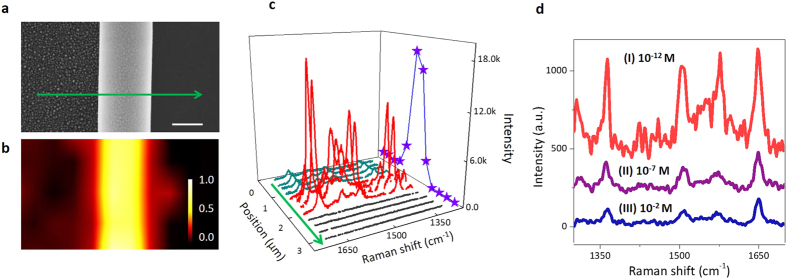
Raman enhancement in a whispering-gallery plasmon nanocavity. (**a**,**b**) SEM image (**a**) and Raman intensity mapping (**b**) of the R6G signal at 1364 cm^−1^ on a rolled-up plasmon nanocavity. The original R6G concentration is 10^−5^ M and the excitation wavelength is 514.5 nm. (**c**) Raman spectra of the line scan along the green arrow marked in a. The blue stars refer to the intensity of 1650 cm^−1^ band extracted from the spectra. (**d**) A comparison of SERS detection limits on Ag NP-decorated plasmon nanocavities (I), undecorated nanotubes (II) and flat silver-NP-decorated nanomembrane (III). The concentrations of R6G solution used are marked in the figure.

**Figure 3:**
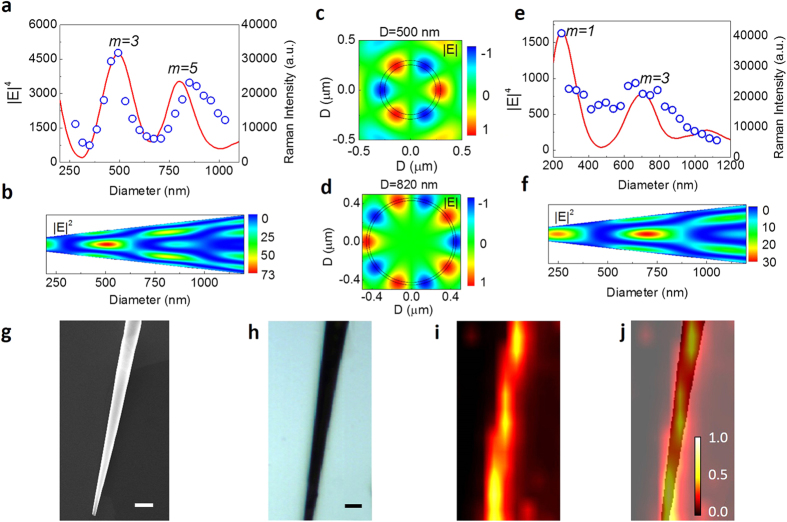
Tunability of WGMs and Raman enhancement in the plasmon nanocavity. (**a**) Relative Raman enhancement of the R6G as a function of plasmon nanocavity diameter using a 514.5-nm excitation. The calculated |E|^4^ (red line) in the cavity wall is plotted together with the measured Raman intensity (blue circles). (**b**) Electric field distribution at 514.5 nm in the plasmon nanocavity with various diameters calculated by FDTD simulation. (**c**,**d**) Calculated field profiles for the resonant cases in plasmon nanocavities with a 514.5-nm excitation, showing the whispering-gallery plasmon cavity modes for the cavity diameter of 500 nm (azimuthal mode number, m = 3) (**c**) and 820 nm (m = 5), (**d**) respectively. (**e**) Relative Raman enhancement of the R6G as a function of plasmon nanocavity diameter using a 632.8-nm excitation. The calculated |E|^4^ in the cavity wall is plotted together with the Raman intensity. (**f**) Electric field distribution at 632.8 nm wavelength of WGMs in the plasmon nanocavity with various diameters calculated by FDTD simulation. (**g**,**h**) SEM image (**g**) and optical microscopy image (**h**) of a conical plasmon nanocavity with diameters varying from 150 to 900 nm. (**i**) Raman intensity mapping of the R6G signal at 1364 cm^−1^ from a conical plasmon nanocavity; R6G concentration is 10^−5^ M; excitation wavelength is 514.5 nm. (**j**) Superposition of images h and i. Scale bar is 1 μm.

**Figure 4:**
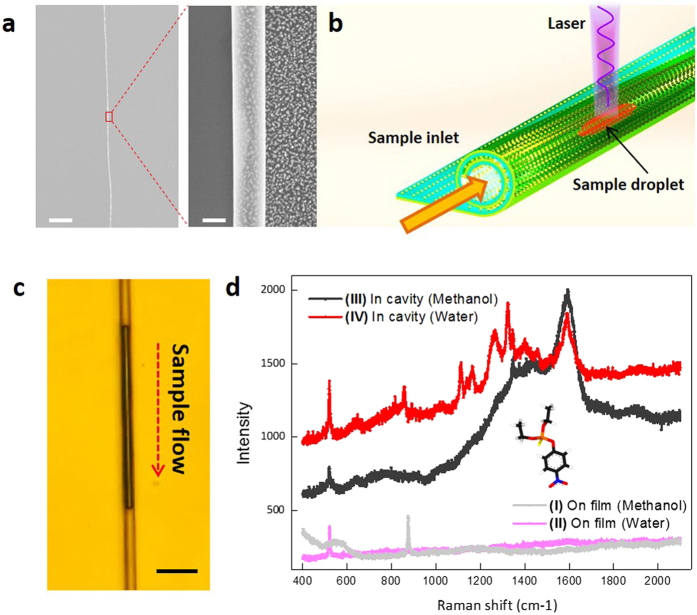
Small volume inspection of pesticide in the plasmon nanocavity. (**a**) A long and uniform rolled-up plasmon nanocavity. Scaler bars: left 10 μm; right 500 nm. (**b**) schematic illustration of the plasmon nanocavity working as an nanoscale optofluidic detector. (**c**) Microscopy image of small-volume pesticide solution pumped in the plasmon nanocavity. Scale bar is 3 μm. (**d**) Measured Raman spectra of parathion. Curve I, on flat Ag NP array using methanol as solvent; curve II, on flat Ag NP array using water as solvent; Curve III, in plasmon nanocavity using methanol as solvent; Curve IV, in plasmon nanocavity using water as solvent. Pesticide parathion concentration is 10^−5^ M.
