# Supplementary material for: Whispering-gallery nanocavity plasmon-enhanced Raman spectroscopy
Source: Sci Rep. 2015 Oct 7;5:15012. doi: 10.1038/srep15012 (PMC4595732; doi:10.1038/srep15012)
Supplement: Supplementary Information [file srep15012-s1.pdf]

Supplementary Notes to

Whispering-gallery nanocavity plasmon-enhanced Raman spectroscopy

Jing Zhang[1,2,#], Jinxing Li[1,3,#], Shiwei Tang[1,#], Yangfu Fang[1], Jiao Wang[1,3], Gaoshan Huang[1], Ran Liu[3], Lirong Zheng[3], Xugao Cui[2] and Yongfeng Mei[1,]*

1. Department of Materials Science, Fudan University, Shanghai 200433, People's Republic of China

2. Department of Light Sources & Illuminating Engineering, School of Information Science &Technology, Fudan University, Shanghai 200433, People's Republic of China

3. School of Information Science &Technology, Fudan University, Shanghai 200433, People's Republic of China

[#] JZ, JL and ST contributed equally to this work.

* Email address: yfm@fudan.edu.cn

1. Refraction index of Ag nanoparticles-air composite layer

As demonstrated in previous studies[1,2], SERS intensity is related to the electric field enhanced by surface plasmon of silver NPs. In the rolled-up plasmon nanotubes, silver NPs are distributed on the inner tube wall of TiO_2/SiO layers. Different from pure oxide nanotubes, material dispersion of both the silver nanoparticles and dielectric SiO/TiO_2 thin films should be considered during the calculation of refraction index. Here, we employed Effective Media Theory (EMT)[3] and Mie scattering[4] methods to calculate the refraction index of Ag nanoparticles-air composite layer as following.

In the range of particle sizes smaller than wavelength, dipole approximation is expected to be adequate when the distances between particles are larger than their diameter, thus we have a dynamic polarizability written as,

$$\alpha = \mathrm{i}\frac{3a^3}{2x^3}a_1 \quad (1)$$

where $x = 2\pi a/\lambda$, $\mathrm{a} = \mathrm{D}/2$ is the radius of the particle. The Mie coefficient a_1 is given by

$$a_1 = \frac{m\psi_1(mx)\psi_1'(x) - \psi_1(x)\psi_1'(mx)}{m\psi_1'(mx)\xi_1'(x) - \xi_1(x)\psi_1'(mx)} \quad (2)$$

Where $\psi_1(x)$ and $\xi_1(x)$ are the Riccati-Bessel functions and $\mathrm{m} = \sqrt{\epsilon/\epsilon_{air}}$ is the ratio of the refraction index of silver NPs to that of air. Assuming that the effective dielectric function of the composite layer is related to the dipole polarizability of NPs by the Clausius-Mossoty equation, we can obtain the following extended Maxwell-Garnett formula,

$$\epsilon_{\mathrm{eff}} = \frac{(a^3 + 2f\alpha)}{\mathrm{a}^3 - f\alpha}\epsilon_{air} \quad (3)$$

where f is the volume fractions of the nanoparticles (f < 0.5).

As the NP size distribution function is regularly log-normal, it can be description as

$$\mathrm{f_{LN}}(D) = \frac{1}{(2\pi)^{\frac{1}{2}} \ln \sigma} \exp\left(-\frac{(\ln D / D_{av})^2}{2 \ln^2 \sigma}\right) \quad (4)$$

where D_{av} is the average diameter of silver NPs and σ is the standard deviation.

The final complex effective dielectric function ϵ_{eff} can be written as

$$\epsilon_{\mathrm{eff}} = \int_0^\infty \epsilon_{eff}(D)\, f_{LN}(D) dD \quad (5)$$

With parameters extracted from our NPs ($\mathrm{f} = 0.2, \sigma = 1.4, \mathrm{D_{av}} = 25\mathrm{nm}$), and dielectric constant of silver described by Drude model (ϵ), the complex effective dielectric function can be obtained as shown in Figure s1, in which the effective refraction index of Ag nanoparticles-air composite layer under the excitation of 514 nm-laser and 633 nm-laser are $\tilde{n}_{514} = 1.49 + 0.02807i$ and $\tilde{n}_{633} = 1.41 + 0.009521i$, repectively.

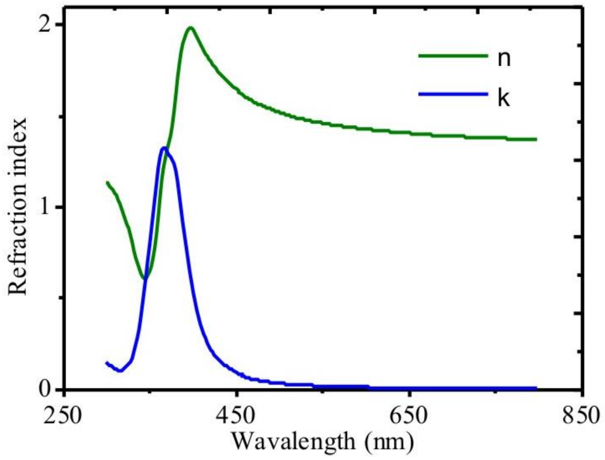

Figure S1 | Refraction index of the silver nanoparticles-air composite layer ($\tilde{\boldsymbol{n}} = \boldsymbol{n} + \boldsymbol{k}\boldsymbol{i}$) based on EMT theory.

2. Calculation of the enhancement factor (EF)

Although there are some uncertainties associated with the EF calculations, it still remains an important and acknowledged way to evaluate the SERS activity of certain substrates.[5-8] Here, we employed a simplified method to estimate the EF as demonstrated following:[9]

$$\mathrm{EF} = \frac{I_{SERS}N_{RRS}}{I_{RRS}N_{SERS}} \quad (6)$$

Where I_{SERS} is the measured SERS intensity on our enhanced substrate (nanotubes with/without silver NPs) and I_{RRS} is the intensity acquired on our referenced substrate (flat nanomembrane without silver NPs). Considering our experimental condition that detection molecules are absorbed on the nanotubes by dropleting R6G solution on the sample and dried in air, the number of detected molecules is calculated by[10]

$$\mathrm{N} = N_A M_{solution} V_{solution} (S_{laser}/S_{substrate}) \quad (7)$$

Where N_A is the Avogadro's number, $M_{solution}$ is the detected solution (R6G) concentration, V the volume of the detected solution and S refers to the detected area. Here, we suppose the detected solution as a cylinder with the diameter of the laser spot (1 μm) and the height of the cylinder equals the height of the total solution before drying.

The EF of SERS intensity on plasmon naotubes approaches the value of 10^{10}, and the whispering gallery modes in nanotubes contribute a value of about 10^5 as calculated in Table S1.

TABLE S1 | Parameters for the calculation of EF on different kinds of substrates

	EF	Intensity/counts		$M_{solution}$ /M	$V_{solution}$ /mL	S_{laser}/ μm²	$S_{substrate}$ /cm²
		1650 cm^{-1}	1364 cm^{-1}				
**Plasmon nanotubes**	~10^{10}	734	691	10^{-12}	0.5	~ π	~ 1
**Nanotubes**	~10^{5}	286	164	10^{-7}	0.5	~ π	~1
**Nanomembrane**	1	162	112	10^{-2}	0.5	~ π	~1

3. Influences of silver nanoparticles on the diameter of nanotubes

Compared to selective removal of the sacrificial layer by chemical wet etching, high temperature annealing shows several advantages. It is calculated that the surface tension of silver NPs promotes a smaller diameter of nanotubes[11]. Experiments are well conducted to compare the diameters of nanotubes with and without nanoparticles as following.

SiO/TiO_2 (5 nm/5 nm) nanomembranes are deposited on the sacrificial layer (polymethyl methacrylate, PMMA) by electron beam deposition, and then the sample is divided into two parts, with one deposited with 3 nm silver layer on the SiO/TiO_2 nanomembranes and the other one acts as the reference. The two samples are annealed

at 600 ^{o}C for 40 s. The statistics for the diameters of the nanotubes are exhibit in Fig. S2. For rolled-up nanotubes with silver NPs, the diameters distribute from 0.3 μm to 1.6 μm, with an average diameter of 1.0 μm. While for the sample without silver NPs, the minimal diameter is 1.3 μm, and the largest one is 2.9 μm, with the average diameter of 1.9 μm, which is about one times larger than that with silver NPs. The experimental results confirm that NPs play an important role in the rolling process of pre-strained nanomembranes and can help the rolling of nanotubes.

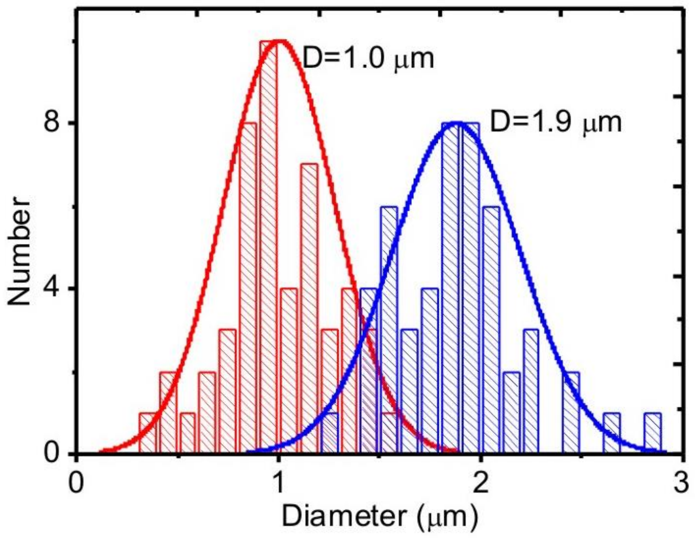

Figure S2 | Diameter distribution of rolled-up nanotubes with/without silver NPs. The statistics is done over 50 nanotubes in a selected region. The red line and columns refer to the results of nanotubes consisting of SiO/TiO_2/silver (5/5/2.5 nm), and the blue line and columns present the nanotubes consisting of SiO/TiO_2 (5/5 nm). Size and distribution of NPs were calculated by the software "ImageJ".

References:

1. Morton, S. M., Daniel W. S., and Lasse J., Theoretical studies of plasmonics using electronic structure methods. *Chem. Rev.* **111**, 3962-3994 (2011).

2. Hao, E., and George C. S., Electromagnetic fields around silver nanoparticles and dimers. *J. Chem. Phys.* **12**0, 357-366 (2003).

3. Kravets, V.G., et al., Plasmonic blackbody: Strong absorption of light by metal NPs embedded in a dielectric matrix. *Phys. Rev. B* **81**, 165401 (2010).

4. Zhan, T.R. et al. Optical resonances in tubular microcavities with subwavelength wall thicknesses. *Appl. Phys. Lett.* **99**, 211104 (2011).

5. Hatab, N. A. et al. Free-Standing Optical Gold Bowtie Nanoantenna with Variable Gap Size for Enhanced Raman Spectroscopy. *Nano Lett.* **10** (12), 4952 (2010).

6. Zhang, L., Lang, X. Y., Hirata, A., & Chen, M. W. Wrinkled Nanoporous Gold Films with Ultrahigh Surface-Enhanced Raman Scattering Enhancement. *ACS Nano* **5** (6), 4407 (2011).

7. Lim, D. K. et al. Highly uniform and reproducible surface-enhanced Raman scattering from DNA-tailorable nanoparticles with 1-nm interior gap. *Nature Nanotechnolofy* **6** (7), 452 (2011).

8. Wells, S. M., Retterer, S. D., Oran, J. M., & Sepaniak, M. J. Controllable Nanotabrication of Aggregate-like Nanoparticle Substrates and Evaluation for Surface-Enhanced Raman Spectroscopy. *ACS Nano* **3** (12), 3845 (2009).

9. Le Ru, E. C., Blackie, E., Meyer, M., & Etchegoin, P. G. Surface enhanced Raman scattering enhancement factors: a comprehensive study. *Journal of Physical*

Chemistry C **111** (37), 13794 (2007).

10. Zhang, L., Lang, X. Y., Hirata, A., & Chen, M. W. Wrinkled Nanoporous Gold Films with Ultrahigh Surface-Enhanced Raman Scattering Enhancement. *ACS Nano* **5** (6), 4407 (2011).

11. Li, J. et al. Dry-released nanotubes and nanoengines by particle-assisted rolling. *Adv. Mater.*, **25**, 3715-3721 (2013).
